# Planning healthy food environments: An analysis of local government municipal public health and wellbeing policy in regional Victoria

**DOI:** 10.1002/hpja.898

**Published:** 2024-07-16

**Authors:** Fiona Dangerfield, Kylie Ball, Virginia Dickson‐Swift, Lukar E. Thornton

**Affiliations:** ^1^ Violet Vines Marshman Centre for Rural Health Research, La Trobe Rural Health School, La Trobe University Bendigo Victoria Australia; ^2^ Institute for Physical Activity and Nutrition, School of Exercise and Nutrition Sciences, Deakin University Waurn Ponds Victoria Australia; ^3^ Department of Marketing, Faculty of Business and Economics University of Antwerp Antwerp Belgium

**Keywords:** food environment, health policy, local government, qualitative study, regional/rural

## Abstract

**Issue Addressed:**

While there has been an increased focus on how local governments can support the creation of healthy food environments through healthy public policy, little is known about how this is reflected in public health policy in regional areas. The aim of this study was to understand how improvements to the food environment are prioritised, implemented and evaluated by local governments in regional Victoria.

**Methods:**

Using a qualitative descriptive approach, content analysis was undertaken of Municipal Public Health and Wellbeing Plans and Council Plans from 10 regional local governments in Victoria, Australia.

**Results:**

Seventeen policy documents were analysed including 10 Council Plans, 6 Municipal Public Health and Wellbeing Plans and 1 Food Systems Strategy. Findings highlight regional public health and wellbeing plans have very few concrete actions in place to improve food environments.

**Conclusion:**

There is an opportunity for Australian regional local governments to include in their public health and wellbeing plans a greater emphasis on supporting healthy food behaviours, and therefore seeking opportunities to improve food environments through clearly aligned priorities, objectives, actions and measures of success.

**So What?:**

Improving the quality of public health and wellbeing plans can support local government to use policy to drive improvements in food environments leading to improved health and wellbeing for regional communities.

## 1 INTRODUCTION

Unhealthy diets are a major contributor to chronic disease.^1^ In many high‐income countries, including Australia, rates of chronic disease are higher among people living in regional areas than those living in urban areas.^2^, ^3^ While the extent to which food behaviours differ between urban and regional areas is unclear, some evidence suggests adults living in regional areas have less healthy food behaviours compared with adults living in urban areas.^4^, ^5^

Food behaviours (i.e., food purchasing, preparation and consumption) are determined by multiple behavioural, social, economic and environmental factors. One theory widely used to help understand these determinants and guide public health research and intervention efforts to improve food behaviours is socio‐ecological theory.^6^ Within this approach, the relationship between people and their environment is argued to comprise an important influence on food behaviours.^7^ The local food environment (i.e., location, type and mix of food outlets) facilitates or limits opportunities to purchase either healthy or less healthy foods.^7^ There is evidence that the characteristics of regional local food environments differ from those of urban food environments with regional areas more often characterised by limited accessibility and availability of, and increased costs of healthier foods.^8^, ^9^, ^10^ In a recent qualitative study, Australian regional residents describe food cost and availability as important factors in determining the type of foods purchased and where they purchase these foods.^11^

Using a socio‐ecological perspective to acknowledge the important role of environments in influencing health behaviours, policy changes shaped by actors across society, including governments, industry and civil society can play a key role in creating environments that support healthy food behaviours.^12^ Internationally, governments at different levels have focused on improving healthy eating through a range of legislative or policy strategies.^12^ More recently, there has been an emphasis among researchers on how local governments may use policy to create and support healthy local food environments in their jurisdictions.^13^, ^14^, ^15^

Australia has three levels of government (federal, state and local). Local government is the level of government closest to the people,^16^ with more than 550 local government areas (LGAs) across Australian states and territories. According to Victorian legislation, the role of a Council is to provide ‘*good governance in its municipal district for the benefit and wellbeing of the municipal community*’ (Section 8 (1)),^17^ which can include but is not limited to matters related to health. The position, influence and connection of local government with their communities provides opportunities to identify public health priorities, and to respond with targeted strategies in collaboration with local communities and organisations to improve health and wellbeing.^18^

Historically, local governments in Australia have been involved in food system activities with traditional areas of focus including food safety, land use zoning for agriculture, horticulture and retail, monitoring food retail premises, and the provision of food in local government facilities and settings.^19^ Recent research suggests some Australian local governments have expanded their focus into climate change and food insecurity^20^; improving Aboriginal nutrition^21^; and creating healthy, sustainable and equitable food systems.^22^, ^23^, ^24^ While recent research has aimed to understand how urban Australian local governments support the creation of healthy food environments^15^, ^25^ very little research has focused on the role local governments may have in creating healthy food environments in regional areas. This is a significant gap, with studies highlighting local policy approaches have the potential to promote and support healthy food behaviours in regional communities.^19^, ^22^

Public health legislation in the Australian states of Victoria,^26^ South Australia^27^ and Western Australia,^28^ require local governments to develop public health plans which provide legislative leverage points for health and wellbeing policies and actions. For example, Victoria's *Public Health and Wellbeing Act 2008* sets out the responsibilities of local governments to protect, improve and promote public health and wellbeing^26^ and all Victorian local governments are required to prepare a Municipal Public Health and Wellbeing Plan that includes goals and strategies to improve the health and wellbeing of their local communities.^26^ This study draws on information from the *Victorian Public Health and Wellbeing Plan 2015–2019* ^29^ and the *Victorian Public Health and Wellbeing Plan 2019–2023*.^30^ Improving healthy eating through collaborative place‐based approaches, such as creating food environments supporting healthy eating, has been identified as a strategic area of focus in both plans.^29^, ^30^ However, it is unknown what priority regional local governments place on improving food environments, and what measures or indicators are used when incorporating action to improve food environments in the policy and planning process. Therefore, the aim of this study was to understand how improvements to the food environment are prioritised, implemented and evaluated by local governments in regional Victoria.

## 2 METHODS

### 2.1 Study design

The present research is placed within a constructivist paradigm and subjectivist epistemology, implying reality is subjectively constructed by researchers and there is a closeness to what is being researched.^31^ In order to investigate the inclusion of food environments in local government municipal public health and wellbeing plans, a qualitative descriptive study design was adopted and qualitative content analysis guided data collection and data analysis.^32^ This approach provided a systematic method for reviewing and evaluating documents to elicit meaning and develop understanding about what was present and not present in the data.

### 2.2 Study setting

Australia is classified into five geographic areas of remoteness (major cities, inner regional, outer regional, remote and very remote areas) based on a measure of relative access to services.^33^ The Australian Statistical Geography Standard classification of inner regional and outer regional areas is comparable to the classification of ‘rural’ used in United Kingdom (UK) and USA studies.^34^, ^35^ Victoria (Australia's second most populous state) is divided into 79 LGAs, each governed by a local government (Council).

Purposive sampling in public health policy analysis at the local government level allows researchers to strategically select documents that address specific research questions, capture diverse perspectives, and provide in‐depth insights into the complexities of public health policy. In Victoria, there are 48 regional and rural council areas across the state contained within 5 Department of Health regions (Barwon South West; Gippsland; Grampians; Hume; Loddon Mallee).^36^ For this study, a sample of 10 LGAs located within one region were selected. The LGAs were chosen to reflect inner and outer regional areas^33^ of varying population size across the region. Demographic characteristics of the LGAs including: remoteness area; population size; median age (years); percentage born in Australia; median weekly household income; percentage unemployed and percentage of population who are obese (BMI ≥30) were reflective of regional Victoria (Table 1).

**TABLE 1:** Characteristics of the 10 regional Victorian local government areas (LGAs) included in study.

	Remoteness area classification^a^	Population^b^	Median age (years)^b^	% Born in Australia^b^	Median weekly household income ($)^b^	% Unemployed^b^	% Adults classified as obese BMI ≥ 30^c^
State of Victoria		5 926 624	37	64.9	1419	6.6	19.3
LGA1	Outer regional	6201	51	84.0	839	4.4	24.5
LGA2	Inner regional	37 061	45	84.3	1081	4.9	25.7
LGA3	Inner regional	12 995	50	82.0	775	8.9	30.6
LGA4	Outer regional	10 549	49	86.5	908	4.8	31.6
LGA5	Inner regional	110 477	39	84.5	1184	6.4	22.4
LGA6	Inner and outer regional	7516	51	79.8	826	5.3	26.5
LGA7	Inner regional	46 100	42	79.8	1638	4.4	15.3
LGA8	Outer regional	53 878	40	79.3	1064	7.3	31.6
LGA9	Inner regional	18 761	49	79.2	1002	5.4	19.1
LGA10	Outer regional	20 584	40	74.7	1094	5.1	28.6

^a^ Australian Bureau of Statistics.^33^

^b^ Australian Bureau of Statistics.^53^

^c^ Department of Health and Human Services.^54^

### 2.3 Data collection

Data collection occurred in four steps.

#### 2.3.1 Step 1

A search of the 10 local government websites was conducted (by FD) between August and October 2020 to identify and download publicly available policy documents that may contain food environment improvement strategies. Documents were included if they were written after 2010, they were the current version, and they were not in draft form. Identified documents included: council plans; annual reports; municipal public health and wellbeing plans; municipal early years plans; smaller town community plans and other policy/planning/strategy documents pertaining to the environment; disability and inclusion; economic; future planning; positive ageing; youth; rural living and active transport.

#### 2.3.2 Step 2

To ensure all relevant policy documents were identified, an additional search of the local government websites was undertaken using a keyword search. Search terms included: *food environment; food access; food availability; food; nutrition; diet; healthy and liveable*.

#### 2.3.3 Step 3

A broad keyword search of the identified policy documents was conducted to determine the relevance of the document in improving food environments. Search terms were: *food; nutrition; diet; eating; overweight; obesity; fruit; vegetable; consumption; supermarket; outlet; shop; market; health; healthy; wellbeing; liveable; liveability; cost; price; access; availability; agriculture; production; produce; socioeconomic; inequality; inequalities; inequity; inequities; disadvantage; car and transport*.

#### 2.3.4 Step 4

Following the keyword search of all policy documents it was found with the exception of one LGA, Municipal Public Health and Wellbeing Plans and Council Plans were the policy documents referencing the promotion or support of healthy eating or healthy food environments. Therefore, the final sample in this study was limited to Municipal Public Health and Wellbeing Plans and Council Plans from the 2017–2021 (inclusive) planning cycle. In addition, one further document was reviewed, as one local government had developed a Food Systems Strategy, which was referred to in their Council Plan.

### 2.4 Description of Council Plans and Municipal Public Health and Wellbeing Plans

Victorian local governments are required to prepare a Council Plan and a Municipal Public Health and Wellbeing Plan every 4 years. The Council Plan provides the strategic direction for the local government, describing objectives, strategies and indicators for monitoring the achievement of objectives for the designated time period.^17^ The Council Plan also provides a description of the local government's initiatives and priorities for services, infrastructure and amenity.^17^ The Municipal Public Health and Wellbeing Plan is the key public health policy document for local government. It must include data relating to the health status and determinants of health for the local community; identify goals and strategies to improve health and wellbeing; and link to priorities outlined in the State Public Health and Wellbeing Plan.^26^ Some local governments create a standalone Municipal Public Health and Wellbeing Plan, while others integrate health and wellbeing planning into their Council Plan.

### 2.5 Data analysis

The content of the Municipal Public Health and Wellbeing Plans and Council Plans was analysed using qualitative content analysis.^32^ Content of the documents were coded (by FD) using an iterative inductive and deductive process.^37^ Prior to coding, the content in the plans were divided into two main sections: the priority setting component and the contextual or background information.

The priority setting component is the section of the plans where local governments outline what they would like to achieve during the planning cycle. Data from the priority setting sections of the Municipal Public Health and Wellbeing Plans and Council Plans were coded into the following categories: priority areas, objectives, action, and indicators for monitoring or evaluation. The contextual information or background component of the plans provide a description of important characteristics of an LGA, which may provide evidence to support the priority setting component. To determine how the priority setting component aligned with state‐based policy and any identified issues within each LGA, content within the contextual or background information of the Municipal Public Health and Wellbeing Plans and Council Plans was deductively coded using the following categories: vision/mission statements; links to state‐based policy; agriculture and food processing; food and non‐alcoholic beverage intake; food security; health status; population demographics; socio‐economic (SE) disadvantage; transport access; and ‘other’ (which included statements specific to an LGA, for example, liveability, place‐based approaches, built environment). Codes were reviewed by all authors during the coding process.

Following coding, a data extraction table was used to record and categorise data into priority setting information and contextual information. For the priority setting information, statements were initially included relating to food, and health and wellbeing more broadly, and then for the final extraction and analysis, statements specifically related to food purchasing and food consumption at an individual and household level, and food access, food availability and food environments at a community level were included. The inclusion and exclusion criteria for the extraction and analysis of information from the priority setting component of the plans are presented in Supplementary Datafile 1 and those for the contextual information component are presented in Supplementary Datafile 2. Once the data were coded, interpretation involved focusing on similarities and differences across categories, examining the positioning of statements within the document to clarify the context and meaning of what was stated, and the presentation of the findings.^37^

Strategies to enhance the rigour of the research included the use of a codebook and extraction tables to provide structure and agreement within the research team about code definitions and categories; a research journal to record personal reflections and description of the research methods assisted in reflexivity; and regular discussions with all authors about meaning and interpretation of findings.^38^ NVivo v12^39^ was used to store, search and code the Municipal Public Health and Wellbeing Plans and Council Plans, and Microsoft Word 2016 was used to develop the data extraction tables. The Standards for Reporting Qualitative Research (SRQR) guidelines^40^ were used to report this study (Supplementary Datafile 3).

## 3 RESULTS

Seventeen policy documents were included in this analysis across the 10 LGAs. Of the 17 documents, 10 were Council Plans (four of which had the Municipal Public Health and Wellbeing Plans embedded within them) and six were individual Municipal Public Health and Wellbeing Plans that were produced in addition to a local government's Council Plan. The final document included for analysis was a specific Food Systems Strategy developed by one local government.

Analysis revealed regional local government Municipal Public Health and Wellbeing Plans contain limited action to improve local food environments. A summary of (i) statements from the contextual or background information, and (ii) food environment statements from the priority setting component, which were identified in the Municipal Public Health and Wellbeing Plans and/or Council Plans is presented in Table 2.

**TABLE 2:** Summary of contextual/background information, priority areas, objectives, action and indicators for monitoring or evaluation related to the food environment identified in regional local government Municipal Public Health and Wellbeing Plans and Council Plans^a^.

Document	Contextual/background information	Priority areas	Objectives	Action	Indicators for monitoring or evaluation
LGA1					
MPHWP integrated into Council Plan	High intake of sugar‐sweetened beverages Prevalence of chronic disease SE disadvantage Accessibility barriers—public transport Retail precincts Place‐based services	Healthy community	‐	‐	‐
LGA2					
MPHWP integrated into Council Plan	Low intake of F&V High intake of sugar‐sweetened beverages Prevalence of chronic disease SE disadvantage	Health and wellbeing	‐	‐	‐
LGA3					
MPHWP	Low intake of F&V High intake of sugar‐sweetened beverages High intake of takeaway meals and snacks Prevalence of chronic disease SE disadvantage	Healthy eating and active living	Support fresh food intake	Promote healthy eating in the workplace. Conduct an audit within Council owned kitchen facilities. Support Street Harvest or any other community groups in establishing or enhancing community gardens (p16)	‐
Council Plan	SE disadvantage Accessibility barriers—public transport, footpaths	Healthy community	‐	Implement Public Health and Wellbeing Plan (p9)	‐
LGA4					
MPHWP integrated into Council Plan	Accessibility barriers—footpaths and roads Liveability	Healthy community	Provide a range of opportunities that promote active and healthy lifestyles and social connectedness	Develop and implement a Healthy Eating Policy across Council, which includes increasing access to public drinking water (p31)	‐
LGA5					
MPHWP	Low intake of F&V Food insecurity Access to and affordability of nutritious food Prevalence of chronic disease SE disadvantage Accessibility barriers—transport Built environments	Healthy community	‐	‐	Proportion of adults, adolescents and children who rate their health as very good or excellent Proportion of adults, adolescents and children who consume sufficient fruit and vegetables Proportion of adults, adolescents and children who consume sugar‐sweetened beverages daily Proportion of adults and children who ran out of food and could not afford to buy more For investigation: Whether to include overweight and obesity measure: proportion of adults, adolescents and children who are overweight and obese (p6)
		Liveability	‐	‐	Average number of days it takes for Council to action food complaints received from members of the public (p7)
Council Plan	Liveability	Wellbeing and fairness	Healthy community	Implement MPHWP (p11)	‐
			Positive wellbeing	Support a localised sustainable food system (p11) Explore opportunities to develop a food policy that coordinates a broad range of themes, including healthy eating and a Food Hub (p25)	‐
LGA6					
MPHWP	Low intake of F&V High intake of sugar‐sweetened beverages Low water intake Prevalence of chronic disease SE disadvantage	Good physical health	Reduce preventable disease	‐	Decreased prevalence rate of type 2 diabetes in adults (p26)
		Protect and promote health	Increase healthy eating	Support the establishment of the Healthy Eating Active Living (HEAL) network (p31)	Increased consumption of fruit and vegetables Decreased proportion of adults/adolescents/children who consume sugar sweetened beverages daily (p31)
Council Plan	SE disadvantage	Liveability	Lifestyle infrastructure	Provide opportunities for the community to develop community gardens (p38)	‐
LGA7					
MPHWP	Low intake of F&V Prevalence of chronic disease SE disadvantage Accessibility barriers—public transport Built environments	Health and wellbeing	Promote healthy eating	Older people: Meal delivery programs, assistance with food shopping and cooking, community meals (p36)	‐
Council Plan	‐	Health and wellbeing	Promote healthy eating	Promote and provide healthy food and drink options across the shire (p8)	‐
LGA8					
MPHWP	Prevalence of poor mental health Commitment to: improving food literacy, knowledge and skills; work in partnership to increase access to nutritious food and the opportunity for people to produce, sell and buy local food; ensuring effective governance of food safety	Health and wellbeing	Promote and support healthy environments and positive health.	Undertake a settings‐based approach to the promotion of healthy eating. Improve the food supply to the priority settings, e.g., workplaces and sporting clubs through targeted partnerships with suppliers and the Victorian Healthy Eating Advisory Service (p20)	‐
Council Plan	Childhood obesity	Healthy community	Community health and wellbeing	Develop and implement Community Health and Wellbeing Plan (p12)	Fruit and vegetable consumption Obesity and prevalence of type 2 diabetes (p12)
LGA9					
MPHWP integrated into Council Plan	‐	Health and wellbeing	‐	‐	Reduction in the proportion of adults who are overweight or obese (p16)
LGA10					
MPHWP	Low intake of F&V High intake of sugar‐sweetened beverages Food insecurity Prevalence of chronic disease SE disadvantage Accessibility barriers—public transport	Healthy eating and active living—Increase food security Desired outcome: Improved access to sufficient, safe and nutritious foods for all communities	Increase food security	Establish Food Security Working Group Facilitate food security forum Develop and implement food security action plan Build food knowledge capacity with community services (p25)	Number of agencies/community members involved Forum held—number of attendees; feedback via survey; engagement in working group/focus areas. Development of action plan. Number of initiatives completed. Structured and ad hoc implementation of capacity building sessions with community services staff; increase services staff knowledge; number of staff engaged; number of cooking programs delivered (p25)
			Increase food literacy	Continue to deliver and/or support food related programs. Continue involvement in the Victorian Healthy Eating Enterprise (VHEE) (p26)	Number of programs delivered and participant attendance. Program effectiveness from initiative specific evaluation. Attend VHEE quarterly meetings and report back to Partnership and Food Security Working Group (p26)
		‐	Encourage access to locally grown produce	Increase community engagement at Community Garden Support edible garden activities in schools and community (p26)	Community member attendance records. Community groups involved. Number of gardens supported, Number of services ordering plants/planting gardens, Number of students engaged in the school garden (p26)
		Healthy eating and active living—Build supportive environments for healthier eating and active living for the whole community Desired outcome: Improved access to sufficient, safe and nutritious foods for all communities	Promote healthy eating	Supermarket tours (p29)	Attendance numbers, qualitative surveys, pre and post (p29)
			Increasing water consumption & decreasing sugary drinks consumption (junior sporting clubs, community wide)	Conduct a needs assessment and develop an action plan in partnership with relevant stakeholders (p28)	Needs assessment and action plan developed (p28)
Council Plan	Liveability	Community enrichment	‐	Review and implement actions from the Public Health and Wellbeing Plan (p19)	‐

*Note*: Page numbers indicating where in the documents the information was sourced are included, however, references are not provided to maintain anonymity of the individual local governments. (‐) indicates there was no mention of this item in the document.

^a^ Some of the text in this table is used verbatim from the publicly available Municipal Public Health and Wellbeing Plans and Council Plans.

### 3.1 Priority setting component of Municipal Public Health and Wellbeing Plans

#### 3.1.1 Priority areas

Each Municipal Public Health and Wellbeing Plan contained a variety of broad priority areas or goals. No local governments identified improving food environments as a specific priority area in their plan. Only two local governments (LGA3 and LGA10) identified improving healthy eating as a priority area in their plan. The remaining eight local governments had more general statements referring to a healthy community or improving health and wellbeing as a priority area in their plans.

#### 3.1.2 Objectives

Four local governments included food‐related objectives in their Municipal Public Health and Wellbeing Plans, with two local governments (LGA6 and LGA7) identifying healthy eating as an objective, and one local government (LGA3) specifically identifying ‘*supporting fresh food intake*’ as an objective. One local government (LGA10) specified a range of food‐related objectives such as increasing food security, increasing food literacy, encouraging access to locally grown produce, promoting healthy eating, increasing water consumption and decreasing sugary drinks consumption.

#### 3.1.3 Actions

Six of the 10 local governments included a food‐related action in their Municipal Public Health and Wellbeing Plans. Examples of action varied across the plans, including ‘*promoting healthy eating in the workplace*’ (LGA3); ‘*developing and implementing healthy eating policy across Council facilities*’ (LGA4); ‘*establishing or enhancing community gardens*’ (LGA3, LGA10); ‘*establish a healthy eating network*’ (LGA6); ‘*improving food supply in priority settings e.g., workplaces*’ (LGA8) and ‘*sporting clubs*’ (LGA8, LGA10); ‘*meal delivery programs, community meals, assistance with food shopping and cooking*’ (LGA7); ‘*food security planning and working group*’ (LGA10); and ‘*supermarket tours*’ (LGA10). The identified actions were mostly broad statements with limited detail describing how the action would be implemented.

#### 3.1.4 Indicators

Indicators for monitoring or evaluation comprised the least frequently included component of the Municipal Public Health and Wellbeing Plans. Only four local governments included indicators in their plans, with no statements in the plans describing where monitoring or evaluation data would be sourced, or how often data would be collected. Two local governments (LGA5 and LGA6) included indicators related to monitoring dietary intake, for example, ‘*consumption of fruit and vegetables*’ and ‘*daily consumption of sugar sweetened beverages*’. LGA9 included a monitoring indicator regarding the ‘*proportion of adults overweight or obese*’, while LGA5 identified the need to investigate the inclusion of such an indicator. LGA10 was the only local government that included evaluation indicators aligned to each action in their plan.

### 3.2 Alignment across priority setting section of plans (priority areas, objectives, action, indicators)

There was variation in the alignment and description of priority areas, objectives, action and indicators for monitoring or evaluation across the Municipal Public Health and Wellbeing Plans. One local government (LGA9) included a priority area of health and wellbeing and an indicator for monitoring or evaluation ‘*reduction in the proportion of adults who were overweight or obese*’, however did not include any food‐related objectives or actions in their plan. There was a lack of detail in describing specifics examples of action for addressing the objectives and priority area, for example, LGA4 included an objective to ‘*provide a range of opportunities that promote healthy lifestyles and social connectedness*’, however, there was only one action included which was to ‘*develop a healthy eating policy across Council*’.

Four local governments incorporated their Municipal Public Health and Wellbeing Plans into their Council Plan, and these local governments had the fewest food‐related statements. LGA1 and LGA2 did not include any food‐related objectives, action or indicators to address the priority areas of healthy community and health and wellbeing, while LGA9 did not include objectives or actions. LGA4 stated an action to ‘*develop a healthy eating policy across Council*’ as an example of promoting healthy lifestyles.

### 3.3 Alignment between contextual information and priority setting components in Municipal Public Health and Wellbeing Plans

The contextual information or background component of the Municipal Public Health and Wellbeing Plans provide a description of important characteristics of an LGA. All Municipal Public Health and Wellbeing Plans included contextual information related to their LGA (Supplementary Datafile 4). The type of information and level of detail provided pertaining to food and nutrition varied across the Municipal Public Health and Wellbeing Plans. Eight local governments identified dietary intake as an issue for their local population with references to low consumption of fruit and vegetables and/or higher consumption of sugar‐sweetened beverages. Food security was identified by two local governments (LGA5 and LGA10). All local governments included statements describing the health status, demographic profile and levels of socio‐economic disadvantage of the population in their municipal area.

For some local governments, there was a mismatch between contextual information and the priority setting component within the plans (Table 2). For example, LGA1 and LGA2 identified poor dietary intake, higher prevalence of chronic disease and socio‐economic disadvantage as important issues for their community, however, there was an absence of any inclusion of food and nutrition in their plans, let alone specific food environment objectives or action. LGA8 included a range of statements in the contextual information of their Municipal Public Health and Wellbeing Plan describing their commitment to ‘*improving food literacy, knowledge and skills across the municipality*’; ‘*work in partnership to increase access to nutritious food and the opportunity for people to produce, sell and buy local food*’; and ‘*ensure effective governance of food safety*’, however, there were limited examples of action to address these commitments in their plan.

### 3.4 Reflection of state policy in Municipal Public Health and Wellbeing Plans

All local governments made some reference to at least one state‐based health or wellbeing policy in their Municipal Public Health and Wellbeing Plans, however, there were no links made between local plans and state‐based plans regarding the food environment. The *Victorian Public Health Act 2008* was the most common state‐based document referred to in the contextual or background information sections of the Municipal Public Health and Wellbeing Plans. All local governments were clear in describing the legislative requirements placed on them under the *Act* to develop a Municipal Public Health and Wellbeing Plan with the aim to improve, promote and protect public health in their municipal district. Nine of the 10 local governments made reference to the *Victorian Public Health and Wellbeing Plan 2015–2019* in their local Municipal Public Health and Wellbeing Plans. While a few local governments outlined it was a requirement to consider the state‐based plan, only three local governments (LGA2, LGA6 and LGA7) explicitly stated they used the state‐based plan to develop the priority areas in their local Municipal Public Health and Wellbeing Plan. Six local governments referred to the *Victorian Public Health and Wellbeing Outcomes Framework 2016* to provide an approach for monitoring, evaluating and reporting progress of their Municipal Public Health and Wellbeing Plan, however, only two plans (LGA5 and LGA6) included specific healthy eating measures (consumption of fruit and vegetables and consumption of sugar‐sweetened beverages). Some local governments referred to additional state‐based documents such as the *Planning and Environment Act 1987* (LGA1 and LGA6), *Local Government Act 1989* (LGA4 and LGA6), *Victorian Climate Change Act 2010* (LGA5 and LGA6) and the *Environments for Health Framework 2011* (LGA7 and LGA10).

### 3.5 Food Systems Strategy

One local government (LGA5) had developed a specific Food Systems Strategy. This document was identified during data collection and was referred to in the local government's Council Plan as an example of an action to support a localised food system. The 10‐year strategy was the first of its kind for the local government with the vision to create a healthy, equitable and sustainable food system, which supports the local economy, culture and community health and wellbeing. ‘*Enabling access to safe, affordable, nutritious and culturally appropriate foods*’ was one key objective. Improving food supply in settings, for example, workplaces, early learning centres, schools, sporting clubs and health care, supporting food relief agencies and programs, and installing water fountains were examples of action to improve food access. In addition, the development of planning controls that may influence the location of convenience and takeaway restaurants, and the development of a food security factsheet incorporating the location of food deserts, were also identified as examples of action within this objective. A second objective was ‘*supporting the growing, producing, sale and cooking of healthy food locally*’. Examples of action aligned with this objective included establishing and supporting community gardens and community cooking programs. All actions in the strategy included generic statements of what success would look like, for example, ‘*water fountains are installed in locations of need*’, however, there was very little detail on specific monitoring or evaluation indicators.

## 4 DISCUSSION

The aim of this study was to understand how improvements to the food environment are prioritised, implemented and evaluated by local governments in regional Victoria. Given the greater burden of diet related disease in regional areas, it is important to extend upon the prior work undertaken in urban areas.^15^, ^25^ Findings from this study highlight regional local governments have very few concrete actions in place to improve food environments in their public health and wellbeing plans. While most local governments identified promoting health and wellbeing as a priority, only two local governments included healthy eating as a priority area in their plans. Similarly, less than half of the local governments included food‐related objectives in their plans. Given Municipal Public Health and Wellbeing Plans and Council Plans are two core strategic documents for Victorian local governments, including food environments as an area of strategic intervention provides the vision and accountability at a higher level to assist in improving health and wellbeing of their communities.

It is well recognised within socio‐ecological approaches^6^ government policy plays an important role in shaping environments that influence a range of behaviours, including healthy food behaviours. Victoria's 2017–2021 Public Health and Wellbeing Plan has documented the importance of ‘*investing in collaborative place‐based approaches to healthy eating and increasing access to healthy food in communities*’ as a strategic action to increase healthy eating.^30^ However, the state plan does not include any other specific food environment statements, nor does it provide clarity around what place‐based approaches may mean. Thus, the absence of focus on food environments in regional Municipal Public Health and Wellbeing Plans may be reflective of the absence of focus at a state level.

Previous research has highlighted that without specific and detailed action, it is difficult for public health and wellbeing plans to be effective strategic planning documents.^18^, ^41^ The current study found identified actions were mostly broad statements with limited detail describing the action, how it would be implemented and who is responsible for the implementation. Often the content of public health and wellbeing plans reflect the balance between community aspirations and available financial resources and expertise within the local government. Assigning shared responsibility for action beyond local governments in local public health policy is particularly important for smaller regional local governments who are often constrained by limited human resources, funding sources and competing priorities.

Importantly, local governments that integrated their public health and wellbeing plan into their council plan included less detail overall, with fewer food‐related statements, which brings into question the ability of these plans to support improved health and wellbeing. If Victorian local governments continue to move away from creating individual public health and wellbeing plans, or as in the case of local governments in other Australian states who are not required to create similar municipal public health and wellbeing plans, there is a possibility of further diluting the priority placed, and action taken to improve regional food environments.

The conceptual framework of community nutrition environments developed by Glanz et al,^42^ which links policy, environmental and individual variables to behavioural eating patterns, can be a useful lens for critiquing the role local government plays in contributing to healthy food behaviour outcomes. It is not reasonable to expect local government policy to contain actions that address all determinants across both individual and environmental levels. Prioritising approaches are necessary. For example, actions improving the built environment may be reasonable targeting the whole population. Alternatively, individual level strategies aimed at those most in need are a useful way to maximise limited resources. Analysis of the public health and wellbeing plans in this study found very few examples of action/strategies reflecting the community (type, location and accessibility of food outlets) or consumer food environment (quality, price, promotion and placement of food within outlets) domains. Reported strategies included actions such as the development of community gardens, provision of community meals, supermarket tours and meal delivery programs. Traditional community food environment responses such as land‐use zoning to influence fast food restaurant locations were not seen. This is often a result of state government legislative frameworks limiting the ability of local governments to influence the type and location of retail food outlets at a local level.^43^ It is not unexpected actions related to the consumer food environment were not observed given these are store owner/manager‐controlled factors and mostly outside the scope of local government influence. There were some examples of actions concentrated in the organisational food environment domain, such as improving food supply in settings such as sporting clubs, workplaces and Council‐run facilities. The prominence of actions in this domain may reflect the increased capacity and expertise of local governments to work in these settings and the emphasis given to improving food supply in priority settings in the *2019–2023 Victorian Public Health and Wellbeing Plan*.^30^

The inclusion of indicators for monitoring or evaluation of success was noticeably absent in the plans, which is consistent with prior research examining Victorian Municipal Public Health and Wellbeing Plans.^41^, ^44^ Three local governments included indicators from the *Victorian Public Health and Wellbeing Outcomes Framework* ^45^ and the *2015–2019 Victorian Public Health and Wellbeing Plan* ^29^ pertaining to individual health status or dietary indicators. However, these provided no detail regarding what environmental changes are required to measure objectives and action, nor how frequently the indicators would be measured, how the data would be collected, or the magnitude of change local governments would like to achieve.

The need for evidence‐based policy to reduce health inequalities in regional Australia has been acknowledged.^46^ Within Municipal and Public Health Wellbeing Plans, evidence can come from a range of sources,^18^ providing the contextual background that informs the development of local priorities and action within the plans. The Victorian *Public Health and Wellbeing Act 2008* states Municipal Public Health and Wellbeing Plans ‘*must include an examination of data about health status and health determinants in the municipal district*’ and must ‘*identify goals and strategies based on available evidence*’ (Section 26(2)(a)&(b)).^26^ Findings from the current study highlight the disconnect between contextual or background information and statements included in the priority setting components of the plans. Furthermore, there was limited recognition in the plans of the wider role the food environment may have in addressing other identified local government priorities such as liveability, social connection, local economy or tourism. Regional local governments are in a unique position to address the broad social determinants of health, by building healthy public policy, often referred to as a Health in All Policies approach.^47^ Despite most plans including statements describing high levels of socio‐economic disadvantage in their municipal area, there were limited examples of equity‐oriented food environment approaches, which are recommended to address broader social determinants of health associated with unhealthy diets.

An emerging area of interest for researchers and policymakers is the role communities or citizens can have in the development of local public health policies and interventions.^48^ Drawing on people's experiences of how they navigate their food environment in everyday life has the potential to provide new insights into the nature of problems and possible solutions.^49^ There are examples of using citizen science methodologies to build public support and create action to promote healthy urban food environments.^50^, ^51^ Similarly, food policy councils, coalitions or alliances, which include non‐government organisations and local community representation, have the potential to identify local food environment needs and advocate for local healthy policy.^52^

### 4.1 Limitations

The Municipal Public Health and Wellbeing Plans and Council Plans included in this study were publicly available on local government websites. There may be local food environment‐related initiatives not presented in the documents included in this study. However, given the comprehensive document search strategy described in the methods, it is unlikely any such existing documents were missed. The translation of what is written in the plans to ‘action on the ground’ was unable to be determined. Discussions with key people involved in the development of the plans may provide valuable insights into the policy development process, including who is involved, skills of those involved, and the process of identifying priorities, objectives, action and measures of success. Finally, data collection and analysis occurred at a single timepoint, with only one cycle of plans used in this study. It would be interesting to examine public health and wellbeing plans over several planning cycles to assess changes over time.

## 5 CONCLUSION

The present findings suggest there is an opportunity for Australian regional local governments to include in their Municipal Public Health and Wellbeing Plans a greater emphasis on supporting healthy food behaviours, and consequently seeking opportunities to improve food environments through clearly aligned priorities, objectives, actions and measures of success. Developing public health plans that are informed by contextual evidence relevant to an LGA, developed in partnership with local communities and closely aligned with state‐based priorities and plans can contribute to the creation of food environments that support healthy food purchasing behaviours for regional people. Regional areas have unique environmental and community needs. Investigating regional local governments policy and planning barriers and supports, such as skills and training required, funding sources, and identifying potential partners as responsible agents for change would be useful next steps for further regional local government food environment research. Improving the quality of public health and wellbeing plans can ensure their creation is not just an exercise to fulfil legislative requirements, but an opportunity for local government to use policy to improve food behaviours leading to improved health and wellbeing for regional communities.

## FUNDING INFORMATION

This work was supported by a Deakin University, Faculty of Health Postgraduate Research Scholarship to Fiona Dangerfield.

## CONFLICT OF INTEREST STATEMENT

The authors have no conflicts of interest to declare.

## ETHICS STATEMENT

Analysis of the publicly available Municipal Public Health and Wellbeing Plans and Council Plans received an exemption from ethical review from Deakin University Human Research Ethics Committee (Project No. 2020‐420).

## Supporting information


**Data S1:** Supporting Information.

## Data Availability

The data that support the findings of this study are available from the corresponding author upon reasonable request.
