# Supplementary material for: Planning healthy food environments: An analysis of local government municipal public health and wellbeing policy in regional Victoria
Source: Health Promot J Austr. 2024 Jul 16;36(1):e898. doi: 10.1002/hpja.898 (PMC11729998; doi:10.1002/hpja.898)
Supplement: Supplementary file 1 — Data S1: Supporting Information. [file HPJA-36-0-s001.docx]

**Supplementary Datafile 1: Inclusion and exclusion criteria for extraction and analysis of information from the priority setting components within local government Municipal Public Health and Wellbeing Plans and Council Plans**

| **Element identified for data extraction and analysis** | **Included** | **Excluded** |
| --- | --- | --- |
| *Priority areas* | | |
| Initial extraction and Final extraction for analysis* | Priority areas consistent with the following themes: healthy community, local economy, built environment, liveable communities | Priority areas consistent with: Council organisation/ management of resources, natural environment |
| *Objectives* | | |
| Initial extraction | Specific reference to: food, health and wellbeing, broader areas that could relate to food access e.g., transport access, liveability statements, social inclusion, local business development, agribusiness, agriculture, built environment |  |
| Final extraction for analysis | Specific reference to: healthy eating, health and wellbeing | Broader areas that could relate to food access e.g., transport access, liveability statements, social inclusion, local business development, agribusiness, agriculture, built environment |
| *Examples of action* | | |
| Initial extraction | Specific reference to: food, health and wellbeing, broader areas that could relate to food access e.g., transport access, liveability statements, social inclusion, local business development, agribusiness, agriculture, built environment |  |
| Final extraction for analysis | Specific reference to: individual/household level - food purchasing, food consumption, community level – food access, availability, food environment | Broader food-based action e.g., oral health, nutrition education |
| *Indicators for monitoring or evaluation* | | |
| Initial extraction | Indicators relating to: food, health and wellbeing, broader areas that could relate to food access e.g., transport access, liveability statements, social inclusion, local business development, agribusiness, agriculture, built environment |  |
| Final extraction for analysis | Indicators specific to: individual/household level - food purchasing, food consumption, community level – food access, availability, food environment |  |

*Initial extraction includes statements relating to food, and health and wellbeing more broadly. Final extraction for analysis includes statements specifically related to food purchasing and food consumption at an individual and household level, and food access, food availability and food environments at a community level

**Supplementary Datafile 2: Inclusion and exclusion criteria for extraction and analysis of information from the contextual/background information within local government Municipal Public Health and Wellbeing Plans and Council Plans**

| **Contextual Element** | **Included** | **Excluded** |
| --- | --- | --- |
| Vision/Mission/Purpose statements | Specific vision/mission/purpose statements | Values and/or principles statements |
| Link to state-based policy | Specific reference to state documents e.g., Acts, policies, plans, frameworks | Statements linking to: national or international documents, partnerships and collaborations with other organisations to achieve health and wellbeing outcomes e.g., Primary Care Partnerships |
| Agriculture & food processing | Specific reference to: role of agriculture and/or food processing in LGA, connection of agriculture and/or food processing to employment and the economy |  |
| Food and non-alcoholic beverage intake | LGA specific statistics | Broader statements (e.g., reference to state or national level statistics) |
| Food security | LGA specific statistics |  |
| Health status | Specific reference to: health and wellbeing, life satisfaction, social inclusion |  |
| Population demographics | Population size, age distribution, gender, ethnicity, household composition | Housing affordability |
| Socio-economic disadvantage | Income, SEIFA, unemployment, education levels (adults) | Education levels (under 18 years) |
| Transport access | Specific reference to: public transport, community transport e.g., community transport enterprises where community volunteers drive the vehicles, transport routes e.g., road freight and passenger traffic, geographic location e.g., distance to major centres |  |
| ‘Other’ element | Statements specific to a LGA e.g., Council commitments, liveability statements, place-based statements, built environment e.g., improving footpaths, roads, streetscapes | Statements linking to: climate change, information provided in Mayor and/or CEO statements, functions of Council services and departments |

**Supplementary Datafile 3: Standards for Reporting Qualitative Research (SRQR) Checklist**

Developed from: O'Brien B. C., Harris I. B., Beckman T. J., Reed D. A., & Cook D. A. (2014). Standards for reporting qualitative research: a synthesis of recommendations. *Acad Med*. 89(9):1245-1251.

| **No.** | **Topic** | **Item** | **Page #** |
| --- | --- | --- | --- |
|  | **Title & Abstract** |  |  |
| S1 | Title | Concise description of the nature and topic of the study identifying the study as qualitative or indicating the approach (e.g., ethnography, grounded theory) or data collection methods (e.g., interview, focus group) is recommended | 1 |
| S2 | Abstract | Summary of key elements of the study using the abstract format of the intended publication; typically includes background, purpose, methods, results, and conclusions | 1 |
|  | **Introduction** |  |  |
| S3 | Problem formulation | Description and significance of the problem/ phenomenon studied; review of relevant theory and empirical work; problem statement | 2-4 |
| S4 | Purpose or research question | Purpose of the study and specific objectives or questions | 4 |
|  | **Methods** |  |  |
| S5 | Qualitative approach and research paradigm | Qualitative approach (e.g., ethnography, grounded theory, case study, phenomenology, narrative research) and guiding theory if appropriate; identifying the research paradigm (e.g., postpositivist, constructivist/ interpretivist) is also recommended; rationale | 4 |
| S6 | Researcher characteristics and reflexivity | Researchers’ characteristics that may influence the research, including personal attributes, qualifications/ experience, relationship with participants, assumptions, and/or presuppositions; potential or actual interaction between researchers’ characteristics and the research questions, approach, methods, results, and/or transferability | 9-10 |
| S7 | Context | Setting/site and salient contextual factors; rationale | 4-6 |
| S8 | Sampling strategy | How and why research participants, documents, or events were selected; criteria for deciding when no further sampling was necessary (e.g., sampling saturation); rationale | 7-8 |

| **No.** | **Topic** | **Item** | **Page #** |
| --- | --- | --- | --- |
| S9 | Ethical issues pertaining to human subjects | Documentation of approval by an appropriate ethics review board and participant consent, or explanation for lack thereof; other confidentiality and data security issues | 2 |
| S10 | Data collection methods | Types of data collected; details of data collection procedures including (as appropriate) start and stop dates of data collection and analysis, iterative process, triangulation of sources/ methods, and modification of procedures in response to evolving study findings; rationale | 7-8 |
| S11 | Data collection instruments and technologies | Description of instruments (e.g., interview guides, questionnaires) and devices (e.g., audio recorders) used for data collection; if/how the instrument(s) changed over the course of the study | 7-8 |
| S12 | Units of study | Number and relevant characteristics of participants, documents, or events included in the study; level of participation (could be reported in results) | 10 |
| S13 | Data processing | Methods for processing data prior to and during analysis, including transcription, data entry, data management and security, verification of data integrity, data coding, and anonymization/ deidentification of excerpts | 8-10 |
| S14 | Data analysis | Process by which inferences, themes, etc., were identified and developed, including the researchers involved in data analysis; usually references a specific paradigm or approach; rationale | 8-10 |
| S15 | Techniques to enhance trustworthiness | Techniques to enhance trustworthiness and credibility of data analysis (e.g., member checking, audit trail, triangulation); rationale | 9-10 |
|  | **Results/findings** |  |  |
| S16 | Synthesis and interpretation | Main findings (e.g., interpretations, inferences, and themes); might include development of a theory or model, or integration with prior research or theory | 10-20 |
| S17 | Links to empirical data | Evidence (e.g., quotes, field notes, text excerpts, photographs) to substantiate analytic findings | 10-20 |

| **No.** | **Topic** | **Item** | **Page #** |
| --- | --- | --- | --- |
|  | **Discussion** |  |  |
| S18 | Integration with prior work, implications, transferability, and contribution(s) to the field | Short summary of main findings; explanation of how findings and conclusions connect to, support, elaborate on, or challenge conclusions of earlier scholarship; discussion of scope of application/generalizability; identification of unique contribution(s) to scholarship in a discipline or field | 20-24 |
| S19 | Limitations | Trustworthiness and limitations of findings | 24 |
|  | **Other** |  |  |
| S20 | Conflicts of interest | Potential sources of influence or perceived influence on study conduct and conclusions; how these were managed | 2 |
| S21 | Funding | Sources of funding and other support; role of funders in data collection, interpretation, and reporting | 2 |

**Supplementary Datafile 4: Summary of contextual or background information included in regional local government Municipal Public Health and Wellbeing Plans and Council Plans**

| **Document** | **Agriculture and food processing** | **Dietary intake** | **Food security** | **Health status** | **Demographic profile** | **Socio-economic disadvantage** | **Transport access** | **Other e.g., Council commitments, liveability statements, place-based statements, built environment e.g., improving footpaths, roads, streetscapes** |
| --- | --- | --- | --- | --- | --- | --- | --- | --- |
| **LGA1** | | | | | | | | |
| MPHWP integrated into Council Plan | √ | √ | X | √ | √ | √ | √ | Built environment: roads and footpaths  Streetscapes & retail: Business precinct development  Place based services: health services |
| **LGA2** | | | | | | | | |
| MPHWP integrated into Council Plan | √ | √ | X | √ | √ | √ | X | Nil |
| **LGA3** | | | | | | | | |
| MPHWP | √ | √ | X | √ | √ | √ | X | Nil |
| Council Plan | √ | X | X | X | √ | √ | √ | Built environment: roads and footpaths |
| **LGA4** | | | | | | | | |
| MPHWP integrated into Council Plan | √ | X | X | X | X | X | X | Built environment: roads and footpaths  Future development: restaurants, improved dining |
| **LGA5** | | | | | | | | |
| MPHWP | X | √ | √ | √ | √ | √ | √ | Improved health, financial and digital literacy  Build community resilience: strengthen community participation and belonging; increase social connection and reduce loneliness  Built environment: streets, footpaths and housing design; access to amenities |
| Council Plan | √ | X | X | X | √ | X | X | Liveability  One Planet Living: local and sustainable food |
| **Document** | **Agriculture and food processing** | **Dietary intake** | **Food security** | **Health status** | **Demographic profile** | **Socio-economic disadvantage** | **Transport access** | **Other e.g., Council commitments, liveability statements, place-based statements, built environment e.g., improving footpaths, roads, streetscapes** |
| **LGA6** | | | | | | | | |
| MPHWP | √ | √ | X | √ | √ | √ | X | Nil |
| Council Plan | √ | X | X | X | √ | √ | X | Urban growth and liveability |
| **LGA7** | | | | | | | | |
| MPHWP | X | √ | X | √ | √ | √ | √ | Access to healthy built places |
| Council Plan | X | X | X | √ | X | X | X | Nil |
| **LGA8** | | | | | | | | |
| MPHWP | √ | √ | X | √ | √ | √ | X | Council commitments:   - Improving food literacy, knowledge and skills across the municipality; - Continuing to work in partnership to increase access to nutritious food and the opportunity for people to produce, sell and buy local food; - Ensuring effective governance of food safety and hygiene preparation practices across the municipality and according to the requirements of the Food Act 1984; - Managing existing townships and areas and planning for new growth areas with the health and wellbeing of the community in mind; - Building on and supporting community spaces that provide opportunities for social connection. (p31) |
| Council Plan | √ | X | X | √ | √ | X | X | Nil |

| **Document** | **Agriculture and food processing** | **Dietary intake** | **Food security** | **Health status** | **Demographic profile** | **Socio-economic disadvantage** | **Transport access** | **Other e.g., Council commitments, liveability statements, place-based statements, built environment e.g., improving footpaths, roads, streetscapes** |
| --- | --- | --- | --- | --- | --- | --- | --- | --- |
| **LGA9** | | | | | | | | |
| MPHWP integrated into Council Plan | √ | X | X | √ | √ | √ | X | Built environment: roads and footpaths |
| **LGA10** | | | | | | | | |
| MPHWP | √ | √ | √ | √ | √ | √ | √ | Nil |
| Council Plan | √ | X | X | X | √ | X | X | Improve amenity and liveability |
